# Radiomics in rectal cancer: current status of use and advances in research

**DOI:** 10.3389/fonc.2024.1470824

**Published:** 2025-01-17

**Authors:** Wei-Qin Huang, Ruo-Xuan Lin, Xiao-Hui Ke, Xiao-Hong Deng, Shi-Xiong Ni, Lina Tang

**Affiliations:** Department of Ultrasonography, Clinical Oncology School of Fujian Medical University, Fujian Cancer Hospital, Fudan University Shanghai Cancer Center, Fuzhou, China

**Keywords:** prognosis, radiomics, rectal cancer, response to neoadjuvant, staging

## Abstract

Rectal cancer is a leading cause of morbidity and mortality among patients with malignant tumors in China. In light of the advances made in therapeutic approaches such as neoadjuvant therapy and total mesorectal excision, precise preoperative assessment has become crucial for developing a personalized treatment plan. As an emerging technology, radiomics has gained widespread application in the diagnosis, assessment of treatment response, and analysis of prognosis for rectal cancer by extracting high-throughput quantitative features from medical images. Radiomics thus demonstrates considerable potential for optimizing clinical decision-making. In this paper, we reviewed recent research focusing on advances in the use of radiomics for managing rectal cancer. The review covers TNM staging of tumors, assessment of neoadjuvant therapy outcomes, and survival prediction. We also discuss the challenges and prospects for future developments in translational medicine, particularly the need for data standardization, consistent feature extraction methodologies, and rigorous model validation.

## 1 Introduction

Colorectal cancer (CRC) is the third-most common cancer globally, accounting for nearly one-tenth of all cancer-related deaths. Rectal cancer (RC) constitutes a substantial proportion of all CRC cases, ranging from 27% to 58% (1–3). The rising incidence of CRC worldwide is attributed to various factors, such as low-fiber and high-fat diets, excessive red meat consumption, and sedentary lifestyles. Furthermore, CRC is often detected at advanced metastatic stages because of the absence of sensitive screening methods or inadequate adherence to screening protocols. In China, the incidence and mortality rates of CRC are on the rise (4), with the epidemiological characteristics of the disease mainly being intermediate and low, constituting approximately 65% to 75% of RC (5).

RC exhibits a propensity for extrabowel infiltration, lymph node involvement, and distant metastasis. In recent decades, the development of therapeutic strategies and multidisciplinary treatments, such as local excision, total mesorectal excision (TME), and neoadjuvant chemoradiotherapy (nCRT), has reduced the local recurrence and distant metastasis rates (6, 7). Colonoscopy, transrectal ultrasound, computed tomography (CT), and magnetic resonance imaging (MRI) are some of the preoperative imaging methods used to evaluate RC. CT, MRI, positron emission tomography (PET), ultrasound, and other imaging techniques are non-invasive and provide information about tumor morphology and some functional aspects. While CT and MRI offer standardized examination views, they are more expensive and time-consuming compared to ultrasound. Ultrasound, on the other hand, is simpler to operate, safer, and offers high reproducibility but is more operator-dependent and subjective compared to MRI.

At present, the widely accepted “gold standard” for pathological staging in the diagnosis of CRC necessitates post-surgery procedures. The accuracy of preoperative imaging diagnosis remains contentious, and there is still no uniform tumor staging standard. Radiomics, a technology based on machine learning methods for the quantitative analysis of medical imaging, can extract information that is not discernible to the naked eye, thereby enhancing the diagnostic accuracy of diseases (8). In this paper, we have systematically described the research and use of radiomics in RC.

## 2 Concepts and process of analysis in radiomics

The concept of radiomics was initially introduced by the American researcher Gillies (9) and further elaborated upon by the Dutch author Lambin (10). Radiomics is based on medical images obtained from modalities such as CT, MRI, PET/CT, or ultrasound and involves the high-throughput extraction of numerous image features related to diseases. This process involves the transformation of medical images into high-dimensional data, the quantification of the characteristic information of various tumors on medical images, and subsequent statistical analysis of these features to create a statistical model data matrix equipped with classification and prediction functions (11, 12).

While computerized diagnostic systems are capable of extracting eight to 20 image features, radiomics utilizes computer algorithms to extract a significantly larger number of quantitative features from medical images, ranging from several hundred to several thousand image features (13). These quantitative imaging features include aspects such as shape, texture, intensity, and spatial distribution, which can provide valuable data about the disease. The information provided about the tumor microenvironment and phenotype is more detailed when compared to laboratory results, clinical reports, and genomic or proteomic analysis.

The workflow of radiomics primarily involves extracting a multitude of multidimensional features from images and applying automated data characterization algorithms. This process converts the imaging data in the regions of interest (ROIs) into spatial data with high resolution and discoverability through quantitative analysis. The workflow encompasses stages such as data acquisition and analysis, image segmentation, feature extraction, and downscaling, as well as model construction and validation (14).

### 2.1 Data acquisition and analysis

The foundation of radiomics is rooted in the collection of clinical medical imaging data. The development and validation of radiomics in CRC involve data from modalities such as CT, MRI, and PET/CT, among others. The variations in scanning instruments and modes result in significant differences in image parameters. Consequently, obtaining standardized medical imaging data has emerged as a pressing concern in current radiomics research.

Radiomics studies begin with the objective of addressing a specific clinical issue, starting with the identification of the ROI. For example, this may involve analyzing images of cancerous tumors in a study on RC to examine their correlation with treatment outcomes. Analysis of lesions and normal tissues involves creating extensive image databases that are capable of storing a vast repository of image data, forming a comprehensive network (15). To address the variations in the parameters of the captured images, it is necessary to gather the datasets required to solve the clinical problem. Notably, image preprocessing emerges as an essential step in radiomics research, encompassing methods such as alignment, denoising, bias field correction, and correction of image inhomogeneity. However, there is a lack of unified standards to guide the selection of preprocessing methods.

### 2.2 Image segmentation

As the data results may be affected by variations in machines or parameters during the acquisition process, it is necessary to standardize the original image. This involves separating the region of interest from the whole image—a process known as segmentation—which is typically required for medical images. ROI segmentation can be classified into manual segmentation, semi-automatic segmentation, and fully automatic segmentation using software. Each method has its own advantages and disadvantages. Manual segmentation is more accurate but less reproducible. On the other hand, automatic segmentation relies on efficient algorithms that help eliminate subjective errors (16). Currently, there is no proven algorithm for automated segmentation of RC. Manual segmentation is used in most radiomics studies pertaining to RC to identify the location and precise boundaries of ROIs.

### 2.3 Feature extraction and dimensionality reduction

Various quantitative features are extracted from the segmented images. These features include shape-based, histogram (first-order), and texture (second-order) features, as well as features related to intensity and perfusion. A large number of image features are extracted, which include the following: (1) shape-based features that describe the morphological characteristics of the ROI, including voxel volume, ROI area, maximum diameter, and so on; (2) first-order features that use a distribution of individual pixel values without considering spatial relationships—these are typically histogram-based methods that reduce the ROI to a single value for the mean, median, maximum, minimum, and homogeneity or randomness (entropy) of the intensities on the image, as well as histograms of the values of skewness (asymmetry) and kurtosis (flatness); and (3) texture features that are used to describe statistical interrelationships between voxels with similar or dissimilar contrast (17). The extracted features must possess the capability to differentiate between various tissues or disease states. In addition, feature selection is necessary to eliminate redundant and irrelevant features in order to enhance the predictive performance of the model.

### 2.4 Model construction and validation

Exploratory and predictive analyses of the extracted features are conducted using statistical analysis methods. Common statistical methods include univariate analysis, multivariate regression, and machine learning algorithms. The creation of predictive models establishes correlations between image features and clinical outcomes, facilitating tasks such as disease diagnosis, prognosis assessment, and prediction of treatment response.

As the number of features extracted from an image is substantial, an excessive number of features can result in overfitting. This necessitates the need for dimensionality reduction (18, 19). Dimensionality reduction methods that are commonly used (20) include support vector machines (SVM), maximum relevance minimum redundancy (mRMR), random forest (RF), and least absolute shrinkage and selection operator (LASSO) regression. Machine learning methods such as decision trees, naive Bayes, k-nearest neighbors (kNN), logistic regression (LR), SVM, bagging, RF, extremely randomized trees, adaBoost, and gradient boosting decision tree (GBDT) are combined to model and optimize dimensionality reduction results. This is done to extract the most informative features and reduce overfitting, ultimately improving the model’s performance and interpretability.

Study quality evaluation and validation: In radiomics research, assessing the quality and reliability of the study is crucial. This includes scrutinizing the consistency of the data, evaluating the robustness of the model, and validating the predictive performance. Various methods that are commonly used for this include cross-validation, external validation, and ROC curve analysis. Once constructed, models are subjected to rigorous evaluation and performance validation in new samples. All models are initially internally validated and subsequently externally validated (validated in multiple centers) as and when conditions permit. A predictive model that lacks a standardized assessment of its performance may not be suitable for clinical decision-making (21). Therefore, quality assessment should ensure the reproducibility of the study as well as validate the model.

## 3 Deep learning-based radiomics

Deep learning-based radiomics differs from traditional radiomics in that the constructed model can automatically learn to extract and select image features and make predictions, enabling a more comprehensive and in-depth exploration of the information in the image.

Currently, the most commonly used methods for image analysis in radiomics include convolutional neural networks (CNN) and sparse auto-encoders (SAE), among others. Deep learning-based radiomics offers several advantages when compared to traditional quantitative analysis methods in radiomics. First, it enables automated feature extraction, thus eliminating the need for manual or traditional methods while improving the efficiency and accuracy of feature extraction. Second, the deep learning model can process and recognize high-dimensional data. Third, after adequate training, the deep learning-based radiomics model demonstrates better generalization ability and stable performance across different datasets and image types. Fourth, deep learning-based radiomics supports the integration of multimodal data, such as combining CT, MRI, and PET images, to provide a more comprehensive disease analysis. Fifth, it allows for the extraction of deeper biological information from clinical images.

Deep learning-based radiomics can effectively identify microstructural changes in tumor tissues and predict the aggressiveness of lesions and patient prognosis. However, there are several challenges in practical applications of deep learning-based radiomics in image analysis, including issues related to model interpretability and limitations in generalization ability. To address concerns about interpretability, it is necessary to enhance the transparency of the algorithms and the verifiability of the results.

## 4 Radiomics in the diagnosis of RC

### 4.1 Preoperative prediction of tumor TNM stage

Before beginning treatment for RC, patients undergo a comprehensive tumor clinical staging process, which involves clinical examination and imaging investigations. The TNM system is a commonly used staging system for RC that evaluates and classifies tumors based on the T (tumor), N (lymph node), and M (distant metastasis) characteristics. Accurate pre-treatment staging is essential for ensuring precise treatment of RC and serves as a crucial indicator for assessing patient outcomes and prognosis (22).

#### 4.1.1 Prediction of the tumor T-stage

Accurate preoperative staging is essential for selecting appropriate treatment methods and minimizing or preventing drug-induced toxic reactions. Radiomics demonstrates significant clinical application value in the context of T-stage classification. Zhou et al. (23) found that the radiomics features of MRI images in RC are the primary influencing factors in distinguishing between T2 and T3 stages of RC. Wang et al. (24) confirmed that texture feature parameters, such as entropy, standard deviation, and homogeneity, in the combined texture analysis of MRI can significantly enhance the preoperative differential diagnostic ability of rectal partially mucinous adenocarcinomas and classic adenocarcinomas accompanied by focal necrosis. Ma et al. (25) conducted an MRI-based T2-weighted (T2w) radiomics study, demonstrating its ability to differentiate between patients with T1 or T2 and those with T3 or T4 RC for the T-staging diagnosis (AUC 0.813, sensitivity 0.933, and specificity 0.925).

Therefore, the use of radiomics in T-staging of RC prior to treatment can assist doctors in accurately assessing the stage of the tumor and provide an important reference for patients to formulate the most suitable treatment plan. The findings of all these studies indicate that certain features extracted from RC imaging are valuable for predicting pathological T-staging preoperatively. This can assist clinicians in selecting appropriate treatment strategies.

#### 4.1.2 Prediction of the tumor N-stage

Accurate assessment of lymph node (LN) status is crucial for treatment planning, predicting local recurrence, and overall survival in patients with CRC. Additionally, radiomics features of RC provide more detailed morphological and anatomical information, aiding in the identification of the metastatic status of the lymph nodes in RC, thereby accurately predicting the risk of lymph node involvement (26, 27). Huang et al. (28) analyzed and modeled the radiomic features extracted from CT images of patients with RC using statistical and machine learning methods. Using multivariate logistic regression, they constructed a nomogram prediction model by combining the radiomic features with clinical data. This model demonstrated satisfactory performance in predicting lymph node metastasis in CRC (AUC 0.736). Another prediction model that was constructed based on the radiomic features of preoperative MRI images of RC achieved an AUC of 0.818–0.94 for predicting lymph node metastasis in RC (29–34).

Ultrasound-based radiomics also holds clinical value for determining preoperative lymph node metastasis in RC. In their study, Pan et al. (20) obtained an AUC value of 0.827 for the test set for diagnosing lymph node metastasis, with a sensitivity of 0.818 and a specificity of 0.750. The findings of Li et al. (35) on the ultrasound-based radiomics features of RC using 3D ultrasonography highlighted the significant value of the random forest model built using these features in preoperatively identifying the lymph node metastatic status in RC. Xian et al. (36) constructed shear wave ultrasound-based radiomics from preoperative ultrasound examinations of 87 patients with RC, achieving a sensitivity of 87.5% and a specificity of 78.8% with 13 ultrastructures that were selected as significant features.

Chen et al. (37) developed a preoperative predictive radiomics for lymph node metastasis based on rectal tumors, lymph nodes, and surrounding tissues obtained from endorectal ultrasound (EUS), CT scans, and shear wave elastography (SWE) of 115 patients with RC, combined with their clinical data. The multiparametric radiomics pattern graph had the highest predictive accuracy for lymph node metastasis, with a consistency index of 0.857. This makes it a useful tool for preoperative prediction of lymph node metastasis, capturing blood supply and stiffness phenotypes. In patients with RC, analyzing the imaging data obtained using radiomics, extracting the imaging features associated with lymph node metastasis status, and subsequently establishing a prediction model can assist clinicians in predicting the risk of lymph node metastasis. Consequently, radiomics analysis of both the primary tumor and lymph nodes can aid in predicting the lymph node status of patients with CRC.

#### 4.1.3 Prediction of the tumor M-stage

The liver is the most likely organ for CRC to metastasize. Liver metastasis in CRC accounts for 75% to 83% of CRC metastases (38). Early and accurate diagnosis of liver metastasis from CRC is crucial for determining the appropriate treatment for patients. Radiomics analysis of RC can yield valuable insights for predicting the presence of liver metastases that are synchronous (already present at the time of diagnosis) or metachronous (occurring after treatment) (39, 40), as well as synchronous metastases to other sites (41).

Machine learning models that are constructed based on CT and MRI image-based radiomics analysis can be used to predict the development of metachronous liver metastases in RC (42). In a study of a radiomics model based on whole-hepatic portal venous phase (PVP) contrast-enhanced CT (CE-CT) images for predicting metachronous liver metastasis (MLM) in RC within 24 months after surgery (43), the AUCs of the training and validation groups of the radiomics model were 0.84 and 0.84, respectively, indicating that the radiomics model for preoperative whole liver PVP CE-CT could predict MLM within 24 months following RC surgery.

Liang (39) conducted a retrospective analysis of MRI image data from 108 patients to develop a model for predicting MLM. The model was based on t2-weighted images and venous phase sequence images, combined with two machine learning algorithms (SVM and LR). The finding was that the combination of baseline rectal MRI-based radiomics and LR yielded the most effective predictions, achieving a sensitivity of 83%. This was done by employing machine learning models that utilize radiomics and clinical risk profiles to forecast the probability of liver metastasis in patients with CRC. Based on this, implementing neoadjuvant radiotherapy and/or more rigorous follow-up protocols for high-risk patients can mitigate the likelihood of their developing MLM.

By extracting high-throughput information from MRI scans, radiomics can be used to predict distant metastasis in locally advanced rectal cancer (LARC) (44). In a multicenter study involving 235 patients who underwent nCRT (45), a predictive pattern graph derived from multiparametric MRI, combined with clinicopathological factors after deep learning, yielded a C-index of 0.775. MRI-based deep learning radiomics has the potential to predict distant metastasis in patients with LARC undergoing nCRT and can help assess the risk of distant metastasis in patients with varying responses to nCRT. These studies investigated the use of radiomics features to analyze CT images or other imaging data for predicting the development of MLM in RC. However, there are only a few studies on the use of ultrasound image-based radiomics for predicting the development of MLM and distant metastasis in RC.

### 4.2 Evaluation of treatment response in RC

According to guidelines, MRI assessment is necessary for locally advanced rectal cancer. For patients with microsatellite stable/mismatch repair proficient (MSS/pMMR), total neoadjuvant therapy (TNT) should be regarded as the initial treatment for patients with low rectal cancer and/or those at high risk. Patients without high-risk factors may consider chemotherapy accompanied by selective chemoradiotherapy (CRT), TNT, neoadjuvant long-course CRT or short-course radiotherapy (RT) based on the degree of response. For patients suitable for TNT, the preferred chemotherapy timing is after radiotherapy. Non-surgical treatment (NOM) can serve as an alternative to total mesorectal excision (TME) for patients with clinical complete response (cCR) after neoadjuvant treatment. For patients with highly microsatellite unstable/mismatch repair deficient (MSI-H/dMMR), immunotherapy is recommended. The immunophenotype and immune cell composition were different in each radiomic assessment group (46). nCRT significantly improves locoregional disease-free survival, negative surgical margins, and complete response rates (47). Clinical complete response (cCR) is achieved in some patients, with the percentage ranging from approximately 15% to 33% (48, 49). The concept of non-surgical treatment has become feasible for patients who show a cCR after nCRT (50), serving as an alternative to conventional surgery (51). However, there is currently no reliable method for diagnosing a complete response.

In this context, radiomics has emerged as a promising tool that can be used as an imaging biomarker to assess response after tumor treatment. In a recent meta-analysis, the sensitivity and specificity of MRI, endorectal ultrasonography, and CT examinations were 95%/31%, 97%/30%, and 96%/21%, respectively (52). Several clinical features have been suggested to increase the likelihood of cCR, including low levels of carcinoembryonic antigen (CEA) (53–56), small tumor size (57, 58), low tumor/nodal stage (59), low histological grade (60), a small range of tumor circumference (61), high hemoglobin levels (53, 60), and a low neutrophil-to-lymphocyte ratio (61).

Radiomics enables a more comprehensive assessment of tumor characteristics than single-image morphology. Mao et al. (62) conducted a study on the combination of 340 radiomics features derived from 216 CT images, along with clinical variables including the distance of the mass from the anal verge, the lymphocyte/monocyte ratio in the blood, and CEA. They developed a prediction model to distinguish between the presence and absence of cCR. The AUCs of the combined model were 0.926 and 0.872 for the training and validation groups, respectively.

Radiomics features inferred from MRI-based T2W images demonstrate the potential to predict cCR (63–67). In a retrospective study by Shin et al. (65), radiological features were extracted from the ROC of T2-weighted images and apparent diffusion coefficient (ADC) graphs of MRI after nCRT using LR to generate three models: T2-weighted, ADC, and T2-weighted and ADC (combined) radiomics models. The AUCs for predicting cCR after neoadjuvant radiotherapy for locally advanced RC were 0.82, 0.79, and 0.82, respectively.

Apart from MRI and CT images, radiomics modeling based on intrarectal ultrasound can be utilized as a pretreatment biomarker to predict the pathological characteristics of RC. Abbaspour et al. (68) analyzed the radiomics features of EUS images from 43 patients with locally progressive RC. Different machine learning methods were used to construct models, and the study found that the machine learning methods (LR and SVM) performed better in radiomics histological features for EUS. The AUC was 0.71 and 0.76 for LR and SVM, respectively, with an accuracy of 70.0% and 71.5%, sensitivity of 69.8% and 80.2%, and specificity of 70.0% and 60.9%, respectively.

The integration of different imaging modalities in radiomics also has significant clinical value in predicting cCR (69–71). In a study, CT and MRI images of 118 cases of RC prior to neoadjuvant chemotherapy were evaluated by Li et al. (69) Based on the performance of different modalities of CT and MRI, including ADC, dynamic contrast-enhanced T1 (DCE-T1) images, and high-resolution T2-weighted imaging (HR-T2WI), imaging features were used to construct a multimodal imaging radiomics model for predicting pathological response. The AUC of the model in the training group and validation group was 0.925 and 0.93, respectively.

Feng et al. (71) developed an integrated radiological-pathological histology prediction system using machine learning. The system is based on three feature sets: radiomics MRI features, pathological nuclear features, and pathological microenvironmental features from a retrospective training cohort. The study was conducted in multiple centers and aimed to predict pathological complete remission. The model predicted cCR with good accuracy (AUC 0.870), 88.8% sensitivity, and 74.0% specificity, which was significantly better than the unimodal prediction model.

Scholars have explored the use of imaging to analyze radiomics features through various modalities such as CT, MRI, PET/CT, and ultrasound images. The aim is to develop a prediction model and corresponding clinical indices to forecast the pathological response of patients with RC following neoadjuvant therapy. However, there are variations in the sample size and methodology across these studies, and as of yet, none of them have undergone validation and confirmation through research for actual clinical applications.

### 4.3 Predicting the prognosis of RC patients

Imaging technologies have also been utilized to predict the survival expectancy of patients with RC, offering valuable insights for treatment selection and patient stratification. Several studies have indicated an association between imaging characteristics and both progression-free survival (PFS) and overall survival (OS). Additionally, clinicopathologic factors and imaging information have been found to be related to the prognosis of patients with locally advanced RC (44, 72–77).

Some histopathologic features, such as extramural vascular invasion (EMVI), degree of differentiation, and perineural invasion (PNI), were associated with a poor clinical prognosis (49). EMVI is a significant factor contributing to a higher risk of recurrence and serves as an independent indicator of a poorer prognosis in RC (78). PNI status was shown to independently predict the local recurrence or progression of RC (79), suggesting that the tumor may have a more aggressive phenotype. It is a key factor in determining whether patients with stage II RC are likely to benefit from nCRT and postoperative adjuvant chemotherapy. High levels of CEA, tumors located less than 5 cm from the anal verge, and a younger age may be associated with a poor prognosis for early-stage rectal cancer after CRT and surgery (80). However, the predictive accuracy of these factors remains low, and there is a need for more precise prognostic factors or predictive models.

Radiomics models have proven clinically valuable in predicting the prognosis of patients with locally advanced RC. Chen et al. (81) demonstrated that radiomics based on combinations of multiple MRI sequences could accurately differentiate between recurrent lesions (LR) at the anastomotic site and non-recurrent lesions. In a multicenter, randomized retrospective study (82), MRI images of LARC treated with nCRT were extracted from 3D MS. A radiomics model for predicting disease-free survival (DFS) was established using a hyperparameter-tuned Random Forest classifier. The predictive value of the radiomics model surpassed that of qualitative parameters. Tumor characteristics were analyzed by extracting variables (t2-weighted, diffusion kurtosis imaging, and enhanced t1-weighted) from preoperative and postoperative multiparametric MRIs (83). Radiomics predictive models were generated based on feature stability and the Cox proportional hazards model, showing predictive potential (C-index ≥ 0.77). Additionally, radiomics feature modeling of CT scans after neoadjuvant radiotherapy (84) enhanced the predictive accuracy of OS from 0.672 when using only clinical features to 0.730 when incorporating radiomics features. This improvement can offer valuable insights for tailoring future treatments for patients with LARC.

## 5 Conclusion and outlook

Radiomics has shown considerable potential in the investigation of RC, covering crucial aspects such as diagnosis, treatment evaluation, and prognosis prediction. Despite significant advancements, there remains a need for multicenter and prospective validation studies to ensure the reliability of its clinical application. Currently, radiomics studies of RC predominantly focus on CT and MRI, with limited research on ultrasound, despite its advantages in terms of ease of use, safety, absence of radiation, and high reproducibility. The fusion and combined analysis of various types of imaging data, including MRI, CT, PET, and ultrasound, is anticipated to offer more comprehensive and accurate information, thereby enhancing the reliability of prognostic assessment and treatment decisions.

Integrating information from radiomics and molecular markers will enable a more comprehensive assessment of the prognostic risk for patients with RC. This approach will provide more accurate guidance for individualized treatment by combining insights from imaging, genetics, and other relevant fields. The use of deep learning and artificial intelligence technologies is crucial in extracting and analyzing large-scale imaging data. These technologies help in identifying potential prognostic predictors and imaging features that can enhance the accuracy of prognostic assessment and personalized treatment.

With radiomics poised for continued growth in the future, certain challenges, such as data standardization, consistent feature extraction, model validation, and clinical feasibility, remain to be addressed. Future studies should aim to address these issues and strengthen the integration with clinical practice to fully harness the potential of radiomics in managing RC. This will also lead to the development of more comprehensive screening and surveillance methods in modern medicine.
